# Transcatheter Closure of an LVOT to LA Fistula After Aortic Bioprosthetic Valve Fracture

**DOI:** 10.1016/j.jaccas.2025.106654

**Published:** 2026-01-21

**Authors:** Ramy Sedhom, Adeba Mohammad, Anas Alani, Purvi Parwani, Antoine Sakr, Jason Hoff, Amr Mohsen

**Affiliations:** Division of Cardiology, Loma Linda University Medical Center, Loma Linda, California, USA

**Keywords:** aortic valve, echocardiography, valve replacement

## Abstract

**Background:**

Left ventricular outflow tract to left atrium (LVOT-LA) fistula is an uncommon but serious complication of transcatheter aortic valve replacement (TAVR), associated with adverse clinical outcomes.

**Case Summary:**

A 78-year-old man with a history of surgical aortic valve replacement, followed by valve-in-valve TAVR, presented with significant dyspnea thought to be due to a valve-in-valve patient prosthesis mismatch. He underwent valve fracture of the TAVR valve. Post–balloon valvuloplasty, the patient developed an LVOT-LA fistula. He presented with worsening exertional dyspnea. Transesophageal echocardiography confirmed the fistula, and a percutaneous transvenous closure using an Amplatzer IV vascular plug was successfully performed. After the procedure, the patient's symptoms markedly improved.

**Discussion:**

Percutaneous closure of an LVOT-LA fistula is technically feasible when performed in experienced centers. Small, low-profile occlusion devices, such as the Amplatzer IV vascular plug, are preferred to minimize the risk of interference with adjacent valvar structures.

**Take-Home Messages:**

Intracardiac shunts are uncommon but serious complications of TAVR. Percutaneous closure is technically feasible when performed at experienced centers.

## Visual Summary

**Figure:**
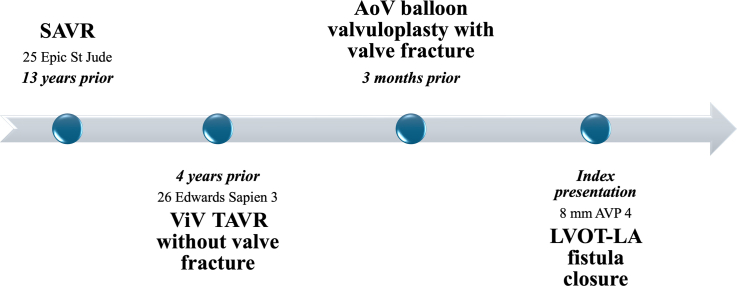

## History of Presentation

A 78-year-old man presented to the structural cardiology clinic for consultation for progressively worsening dyspnea with exertion and extreme fatigue. He had a history of surgical aortic valve replacement, followed by valve-in-valve transcatheter aortic valve replacement (ViV TAVR) 3 years prior. This was followed by bioprosthetic valve fracture of ViV TAVR, which was complicated by a left ventricular outflow tract (LVOT) to left atrium (LA) fistula. He had improvement in heart failure symptoms, but subsequently developed recurrent dyspnea. On examination, the patient was noted to have jugular venous distention, decreased bibasilar lung sounds, and 1+ bilateral pitting edema of the lower extremities.

## Past Medical History

**Figure 1:**
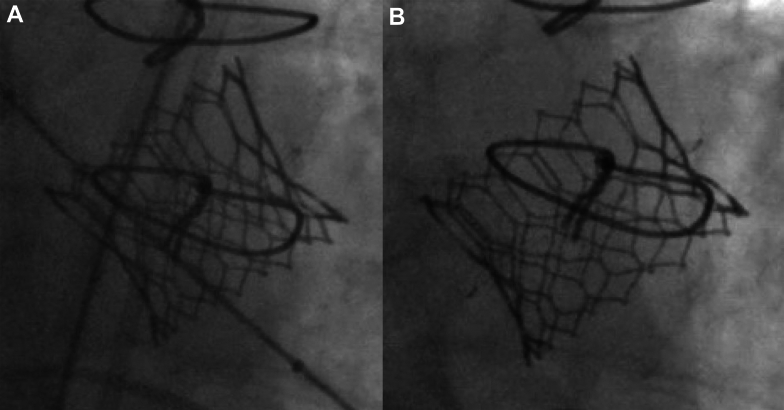
Fluoroscopic Images of Balloon Valvuloplasty (A) Pre–balloon valvuloplasty transcatheter aortic valve replacement (TAVR) valve. (B) Post–balloon valvuloplasty TAVR valve.

The patient has a history of aortic stenosis status-post 25 mm St Jude Medical Epic heart valve (St Jude Medical, Inc) (true internal dimension 21 mm) 13 years prior, followed by ViV TAVR with a 26-mm Edwards SAPIEN 3 bioprosthetic valve 9 years later because of structural valve degeneration. ViV TAVR was performed without valve fracture. Three months before the current presentation, the patient underwent balloon valvuloplasty with a 25-mm True balloon (4 mm greater than the internal diameter of the patient's valve; Bard Vascular Inc) of the TAVR valve with valve fracture for a severe patient-prosthesis mismatch (Figure 1). Balloon valvuloplasty was complicated by an LVOT-LA fistula seen in mitral-aortic intervalvular fibrosa with supra-annular mitral regurgitation identified immediately after bioprosthetic valve fracture, with a plan for watchful waiting because the patient was asymptomatic at the time. He also had decompensated heart failure with a left ventricular ejection fraction of 40% to 45%, moderate functional tricuspid regurgitation, hypertension, hyperlipidemia, chronic obstructive pulmonary disease, and patent foramen ovale.

## Differential Diagnosis

The differential diagnosis for dyspnea includes increased shunting from the LVOT-LA fistula, worsening heart failure, worsening aortic valve function, or primary lung pathology.

## Investigations

**Figure 2:**
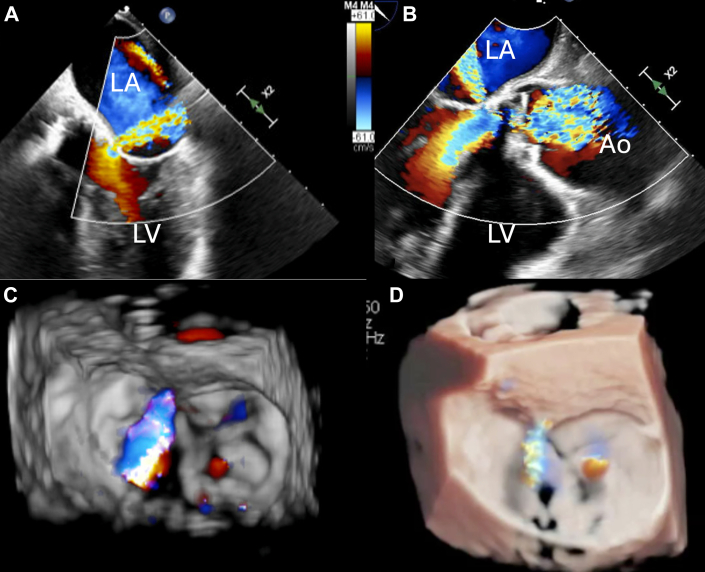
Transesophageal Echocardiogram (A and B) LVOT-LA fistula via the zone of mitral-aortic intervalvular fibrosa (fibrous aortomitral curtain). (C and D) Three-dimensional rendering of the LVOT-LA fistula. Ao = aorta; LA = left atrium; LV = left ventricle; LVOT = left ventricular outflow tract.

Transesophageal echocardiography (TEE) was performed to further understand the valvar pathology and fistula. TEE revealed an LVOT-LA fistula via the zone of mitral-aortic intervalvular fibrosa or fibrous curtain below the posterior aortic cusp, close to the lateral trigone, with at least moderate supra-annular mitral regurgitation with 3-dimensional vena contracta of 0.35 cm^2^ and 2-dimensional vena contracta of 0.4 cm (Figure 2). There was trace mitral regurgitation through the native valve. The mean gradient across the bioprosthetic aortic valve was 11 mm Hg, with no paravalvular leak.

## Management

**Figure 3:**
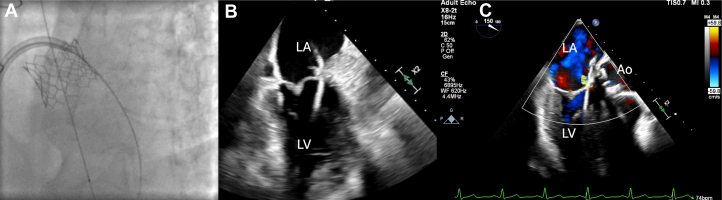
Crossing the Fistula (A) Angled glide wire advanced through the fistula via transseptal puncture and LA on fluoroscopy. (B and C) TEE view of angled glide wire through the fistula into the LV. TEE = transesophageal echocardiographic; other abbreviations as in Figure 2.

**Figure 4:**
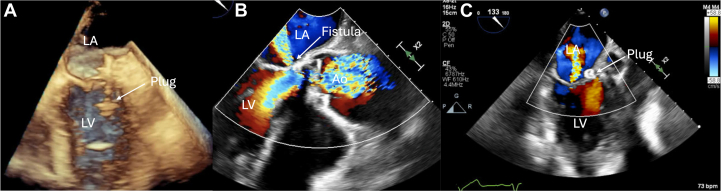
Deployment of the Amplatzer Plug (A) TEE view of the Amplatzer plug in the appropriate position with deployment of the distal disc. (B) TEE view showing the LV-LA fistula. (C) Appropriate position of the device with no residual leak on TEE. Abbreviations as in Figures 2 and 3.

The patient was evaluated by a multidisciplinary approach, with input from structural cardiology and cardiac imaging teams. Closure was indicated because of symptomatic heart failure from a hemodynamically significant LVOT-to-LA shunt, with elevated left ventricular end-diastolic pressure on invasive assessment. Given the patient's high surgical risk and favorable anatomy on TEE, percutaneous transseptal closure with a low-profile device was the preferred approach. A 10-F short sheath was placed in the right common femoral vein. A mid and posterior transseptal puncture under TEE guidance was performed using the standard technique to allow for maximum maneuverability of the wire. The catheter was advanced to the LA. Heparin anticoagulation was administered, and the activated clotting time was maintained within the therapeutic range. A telescoping system of an 8.5-F Agilis steerable guide catheter (Abbott), a 5-F multipurpose diagnostic catheter, and an angled glide wire were used to cross the paravalvular defect from the LA. The wire and the multipurpose catheter were advanced to the left ventricle (Figure 3). An 8-mm Amplatzer IV vascular plug (AVP IV) was positioned in the defect and then released (Figure 4A). Proper positioning was confirmed by fluoroscopy and TEE. Echocardiography demonstrated resolution of the perivalvular leak (Figures 4B and 4C).

## Outcome and Follow-Up

The patient's exertional dyspnea and heart failure symptoms improved significantly.

## Discussion

Iatrogenic intracardiac shunts are an uncommon clinical entity. It has been most frequently reported in association with surgical interventions involving the aortic root and/or aortic valve.^1^ Nonetheless, fistula formation has also been reported after TAVR, where it is associated with increased morbidity and elevated mortality rates, particularly in the presence of heart failure. Contributing risk factors may include extensive perivalvular calcification, aggressive postdilatation, and low implantation. When such fistulous communications occur, they most frequently involve the interventricular septum or extend from the aortic root to the right ventricle. Involvement of the LA is rare.^2^ In the present case, the need for balloon angioplasty and valve fracture of a previously deployed ViV due to a patient-prosthesis mismatch likely contributed to the development of this complication.

The clinical manifestation of such fistulas is typically dependent on the magnitude of left-to-right shunting, as reflected by the pulmonary/systemic flow ratio. Although many patients remain asymptomatic, approximately 50% may develop symptoms of heart failure, hemolytic anemia, or embolic events.^2^^,^^3^ In the present case, the patient had a left-to-left shunt and experienced a recurrence of heart failure symptoms after an initial period of clinical improvement after TAVR.

Conservative management may be appropriate for asymptomatic patients. However, closure of the shunt is indicated in cases with progressive heart failure, right ventricular dilatation, or an increasing pulmonary/systemic flow ratio. Given that these patients are often high-risk surgical candidates, transcatheter closure represents a preferable therapeutic alternative.^2^ In this case, the patient was at high surgical risk because of advanced age and history of valve surgery.

Currently, there are no percutaneous devices specifically designed for the closure of iatrogenic intracardiac shunts after TAVR.^2^ However, percutaneous closure techniques using Amplatzer devices, coil embolization, and covered stents have been reported in the literature.^4^ Small, low-profile occlusion devices are preferred to minimize the risk of interference with adjacent valvar structures and preserve valve function.^2^ The AVP IV is a low-profile nitinol-based embolization device designed for use with 0.038-in guidewire-compatible diagnostic catheters. It is available in device diameters ranging from 4 to 8 mm. The device is mounted on a 155-cm-long polytetrafluoroethylene–coated delivery wire, secured via a stainless steel microscrew mechanism. This configuration enables controlled deployment of the plug by counterclockwise rotation of the delivery cable using the provided torque device. The plug can be recaptured and repositioned before the final release, allowing for optimal placement.^5^ In our case, the defect was small in size and in close proximity to both the native mitral and transcatheter aortic valve leaflets. An 8-mm AVP IV was used because it can be deployed through any diagnostic catheter that can accommodate a 0.035-inch guidewire, such as a 4-F multipurpose catheter. In addition, because of the small size of the discs, there was no interaction between the device and the leaflet motion of both valves.

## Conclusions

LVOT-LA fistula is an uncommon but serious complication of TAVR, associated with adverse clinical outcomes. Percutaneous closure is technically feasible when performed in experienced centers. The use of TEE for both preprocedural planning and intraprocedural guidance is essential to ensure procedural success. Small, low-profile occlusion devices, such as AVP IV, are preferred to minimize the risk of interference with adjacent valvar structures.

**Visual Summary:**
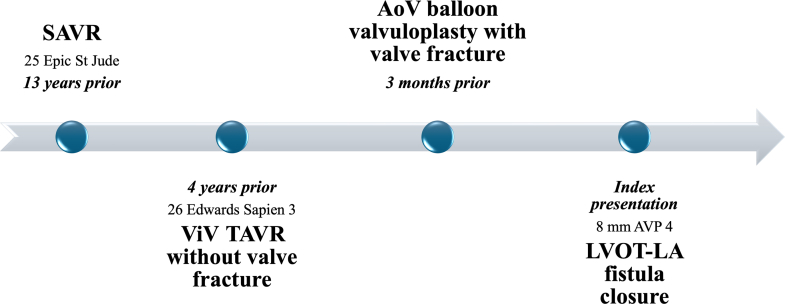
Case Timeline AoV = aortic valve; AVP 4 = Amplatzer IV vascular plug; LA = left atrium; LVOT = left ventricular outflow tract; SAVR = surgical aortic valve replacement; TAVR = transcatheter aortic valve replacement; ViV = valve-in-valve.

## Funding Support and Author Disclosures

The authors have reported that they have no relationships relevant to the contents of this paper to disclose.

**Take-Home Messages**

• Intracardiac shunts are uncommon but serious complications of transcatheter aortic valve replacement.
• Percutaneous closure is technically feasible when performed at experienced centers.

## References

[1] Dayco J.S., Bell K., Alhusain R., Awadelkarim A., Baciewicz F., Cardozo S. (2023). Left ventricular outflow tract to left atrium fistula as a complication of aortic root repair. IHJ Cardiovasc Case Rep.

[2] Rojas P., Amat-Santos I.J., Cortés C. (2016). Acquired aseptic intracardiac shunts following transcatheter aortic valve replacement: a systematic review. JACC Cardiovasc Interv.

[3] Sammour Y., Chawla S., Tsutsui R.S., Patel J., Harb S., Kapadia S. (2021). Transcatheter closure of left ventricular outflow tract–to–left atrium fistula. Case Rep.

[4] Jainandunsing J.S., Linnemann R., Bouma W. (2019). Aorto-atrial fistula formation and closure: a systematic review. J Thorac Dis.

[5] Lopera J.E. (2015). The amplatzer vascular plug: review of evolution and current applications. Semin Intervent Radiol.

